# A survey on the relationship between serum 25-hydroxy vitamin D level and tumor characteristics in patients with breast cancer

**Published:** 2016-01-01

**Authors:** Ghasem Janbabai, Ramin Shekarriz, Homa Hassanzadeh, Mohsen Aarabi, Seyyedeh Samaneh Borhani

**Affiliations:** 1 Associate Professor, Medical Oncologist-Hematologist, Gastrointestinal Cancer Research Center, Imam Khomeini Hospital, Mazandaran University of Medical Sciences, Sari, Iran; 2 Assistant Professor, Medical Oncologist-Hematologist, Gastrointestinal Cancer Research Center, Imam Khomeini Hospital, Mazandaran University of Medical Sciences, Sari, Iran; 3 Internist, Department of Internal Medicine, Imam Khomeini Hospital, Mazandaran University of Medical Sciences, Sari, Iran; 4 Assistant Professor, Epidemiologist, Mazandaran University of Medical Sciences, Health Sciences Research Center, Sari, Iran; 5 General Physician, Gastrointestinal Cancer Research Center, Mazandaran University of Medical Sciences, Sari, Iran

**Keywords:** Breast cancer, Vitamin D, Tumor stage, Menopausal status

## Abstract

**Background:** In recent years, epidemiologic and laboratory studies have implied that vitamin D deficiency has a role in the pathogenesis of breast cancer. It has shown that vitamin D can prevent tumor progression induced by carcinogens and inhibit the carcinogenic effects of high fat diet on breast tissue and growth of tumor cells. This study aimed to evaluate serum 25-hydroxyvitamin D level and its role in relation to tumor characteristics and different stages of disease in women with breast cancer.

**Subject and Methods:** This is a cross-sectional study on 200 patients with breast cancer at different stages of the disease. Information on age, menstrual status, BMI and tumor characteristics were recorded. Serum 25-hydroxy vitamin D concentrations were measured by radioimmunoassay (RIA). Mean and standard deviation were used to describe the data. Meanwhile, T-test and sum of squares test were used to analyze the data. P<0.05 was considered significant.

**Results:** Among 200 patients, 47 (23.5%) had severe vitamin D deficiency, 75 (37.5 %) had mild vitamin D deficiency and 78 (39.0 %) had sufficient vitamin D levels. There was a significant correlation between low vitamin D levels and advanced stage of breast cancer, particularly in postmenopausal patients.

**Conclusion:** It seems that lower levels of vitamin D accompany worse clinicopathologic features. Thus, treatment of vitamin D deficiency in postmenopausal patients might be of great benefits.

## Introduction

 Vitamin D is a fat-soluble vitamin produced in the body.^1^ Its main function is increasing calcium absorption and contribution to bone mineralization. In recent years, epidemiologic and laboratory studies have implied that vitamin D deficiency has a role in the pathogenesis of breast cancer. In vitro studies have shown that 1, 25 (OH) vitamin D inhibits proliferation of breast cancer cells and makes them more differentiated;^2^ also it seems to induce apoptosis.^3^ Experiments on animals have shown that dietary vitamin D can inhibit carcinogenic effects of a high-fat diet on breast tissue. Moreover, 1, 25 (OH) vitamin D and its analogs can inhibit the growth of breast cancer cells and prevent tumor progression induced by carcinogens.^4^^,^^5^^,^^6^ Although 1, 25 (OH) vitamin D is the active vitamin D metabolite; its production is carefully tuned.^7^ Serum 25-OH vitamin D concentration is more correlated with the amount of vitamin D acquired from diet and sunlight exposure and it is a better indicator of the body vitamin D status.^7^^-^^9^ This study aimed to evaluate serum 25-hydroxyvitamin D level and its role in relation to tumor characteristics (local invasion, lymph node involvement, and metastasis) and menstrual status of the patients at Bagheban oncology clinic in Sari.

## SUBJECTS AND METHODS

 This was a cross-sectional study on 200 patients with breast cancer at different stages of the disease. After obtaining informed consent from the subjects, a questionnaire including information on age, parity, menstrual status (pre-menopause, menopause), BMI, and tumor characteristics was completed for each patient. The following factors were considered as exclusion criteria: hepatic or renal failure, metabolic bone disease, malabsorption and recent consumption of vitamin D (patients who had received oral vitamin D in recent two weeks, or vitamin D injection in recent 6 months). Blood samples were obtained from all patients. Serum 25-hydroxyvitamin D concentrations were measured using radioimmunoassay method (RIA) by a Japanese Hitachi analyzer and Elecsys casts. Serum calcium, phosphorus and alkaline phosphatase were measured by standard laboratory method, using Roche kits made in US and Cobas Integra 400 plus analyzer. Regarding the adequacy of vitamin D, the patients were classified into three groups: severe deficiency (<10 ng/ml), mild deficiency (10-25 ng/ml) and no evidence of deficiency (25-80 ng/ml).^10^

Data were analyzed using the SPSS 16.0 software. Mean and standard deviation were used to describe the data. Meanwhile, T-test and sum of squares test were used to analyze the data. P-value less than 0.05 were considered statistically significant. Analysis of variance was used for comparison of more than two groups and analysis of covariance was employed for adjustment of any covariates. This work was approved by the local clinical research ethics committee (No: 92/3/8).

## Results

**Figure 1:**
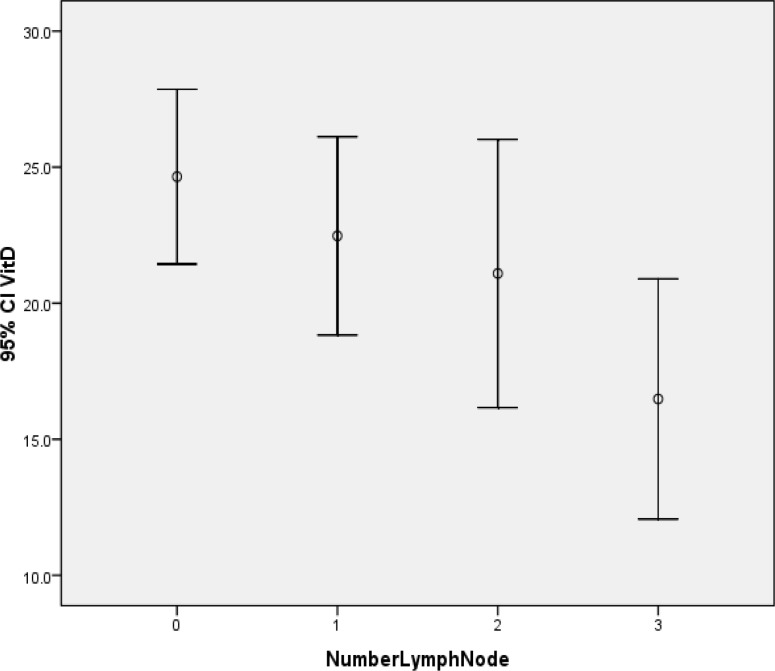
Mean Vitamin D concentration and number of involved lymph nodes

 Mean concentration of serum vitamin D was 22.58 ± 14.21 ng/ ml in 200 subjects, 47 patients (23.5 %) had severe vitamin D deficiency, 75 patients (37.5 %) had mild vitamin D deficiency and 78 patients (39.0 %) had sufficient vitamin D levels in serum. 40 patients (20%) had tumors with histologic grade 1, 128 patients (64 %) had grade 2 and 32 patients (16%) had grade 3 tumor. Evaluation of hormone receptors revealed that 144 (72%) patients were ER+ (estrogen receptor) and 127 (63.5 %) of whom were PR+ (progesterone receptor). Also, one hundred and forty-nine patients (74.5 %) were HER-2 + (human growth factor receptor 2). Lymph node involvement was observed in 111 patients (55.5%), and 20 patients (10%) had distant metastasis. Patients with distant metastases had significantly lower serum vitamin D levels compared to patients without distant metastasis (12.22 ± 2.28 ng/ ml vs. 23.7 ± 1.06 ng/ ml, respectively; P-value = 0.001). Serum vitamin D level in patients with lymph node involvement was lower than patients without lymph node involvement, but the difference was not statistically significant. (1.24 ± 20.91 ng/ ml vs. 1.61 ± 24.65 ng/ ml, P-value = 0.064).The comparison of vitamin D levels in patients with different number of involved lymph nodes, using analysis of variance, showed no significant difference among the 4 groups. (P-value= 0.098)(Figure 1).

**Figure 2:**
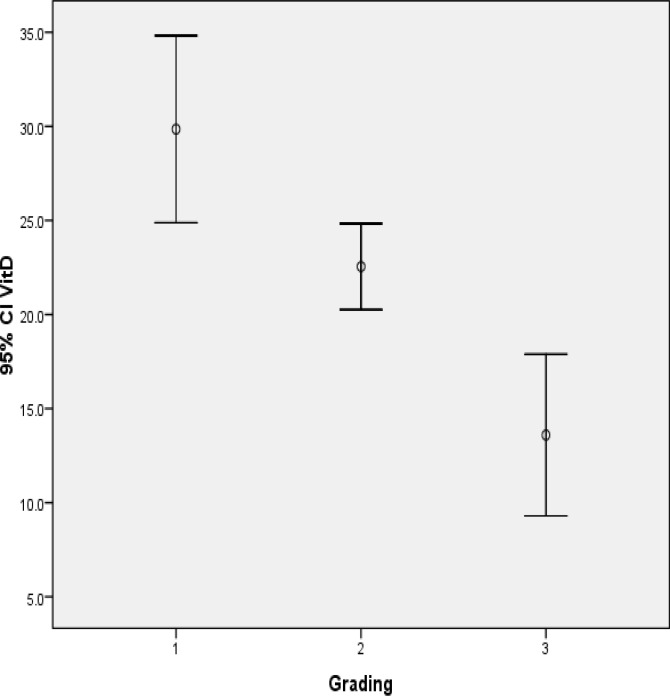
Distribution of mean serum Vitamin D level according to tumor grade

**Figure 3:**
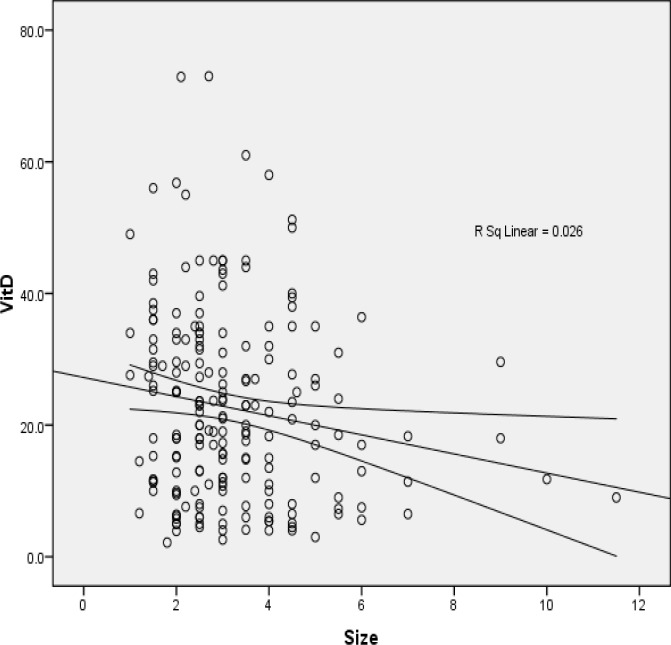
Correlation between serum Vitamin D level and tumor size

Patients with higher tumor grade had lower levels of vitamin D compared to patients who had lower grade. This difference was significant by analysis of variance (P value < 0.0001) (Figure 2). A significant negative correlation was found between tumor size and levels of the vitamin D. Patients with greater size of tumor had lower level of the vitamin D (R = -0.163, p-value = 0.021) (Figure 3). Vitamin D level was slightly different between hormone receptor-positive and hormone receptor-negative patients, but it was not statistically significant. Serum vitamin D level in premenopausal patients was less than postmenopausal ones (14.06 ± 21.54 ng/ ml vs. 14.35 ± 23.69 ng/ ml) and this difference was not statistically significant (P-value = 0.285). Vitamin D level and its association with histologic and prognostic features of breast tumors were also evaluated and compared in premenopausal and postmenopausal patients. The relationship between vitamin D level and disease stage in the two groups (before and after menopause) is shown in Table 1.

Vitamin D levels show no significant difference in premenopausal and postmenopausal patients regarding hormonal receptor and HER2 status (Table 2). The relationship between vitamin D levels and lymph node involvement and also stage of the disease in patients before and after menopause are shown in Table 3 and Table 4, respectively. A significant relationship was observed between low levels of vitamin D and stage of the disease and lymph node involvement only in the postmenopausal patients. After conducting a multivariate analysis, it was revealed that the difference between levels of vitamin D in the patients with early and advanced stages of breast cancer still remained statistically significant (Table 5).

## Discussion

 The protective effect of vitamin D against breast tumor progression was introduced first time by Garland in 1980^22^ and during the following years many studies have demonstrated this association.^15^^,^^16^^,^^23^^,^^24^ In recent years, researchers have focused on the relationship between vitamin D levels and breast cancer prognostic factors such as tumor size, histologic grade and stage of the disease, lymph node involvement, hormone receptor status and metastasis. Different studies have found different and sometimes contradictory results.^20^^,^^21^^,^^25^ In this study, serum vitamin D levels in 200 patients at different stages of breast cancer was evaluated. The mean serum 25-hydroxyvitamin D level was 22.58 ± 14.21 ng/ml. Vitamin D level was normal in 39% of patients, while 37.5% and 23.5 % had mild and severe deficiency, respectively. The results of the study in Iran and the Middle East revealed that the incidence of vitamin D deficiency was between 30% - 80 %, compatible with our findings.^11^^-^^14^ In the present study, serum 25-hydroxyvitamin D level had a significant inverse association with metastatic breast cancer. Low levels of vitamin D were also significantly associated with advanced stages of the disease, tumor size and grade, while no significant correlation was observed between serum 25-(OH) vitamin D and lymph node involvement or hormone receptor status. Of 200 patients, 96 (48%) were postmenopausal and 104 (52%) were premenopausal.

**Table 1:** Relationship between vitamin D level and disease stage according to menopausal status

**Menopausal ** **status**	**Disease ** **stage**	**Mean vitamin D ** **level (ng/ml) ± SD**	**p-value**
**Premenopause**	Early	22.983 ± 14.8852	0.093
	Advanced	17.819 ± 11.0729	
**Postmenopause**	Early	26.473 ± 14.2326	0.007
	Advanced	18.144 ± 13.1192	

**Table 2:** The relationship between vitamin D level and hormonal receptor status in the two groups (before and after menopause)

**Menopausal ** **status**	**Hormonal ** **receptor status**	**Mean vitamin ** **D level (ng/ ml) ** **± SD**	**p-value**
**Premenopause**	Estrogen receptor negative	19.70 ± 10.580	0.421
	Estrogen receptor positive	22.22 ± 15.159	
	Progesterone receptor negative	20.63 ± 9.933	0.653
	Progesterone receptor positive	21.97 ± 15.670	
	HER2 negative	19.85 ± 13.574	0.449
	HER2 positive	22.20 ± 14.291	
**Postmenopause**	Estrogen receptor negative	21.48 ± 13.454	0.410
	Estrogen receptor positive	24.48 ± 14.736	
	Progesterone receptor negative	23.46 ± 14.799	0.89
	Progesterone receptor positive	23.87 ± 14.163	
	HER2 negative	23.90 ± 19.083	0.940
	HER2 positive	23.64 ± 12.783	

**Table 3:** The correlation between vitamin D level and the number of involved lymph nodes

**Menopausal ** **status**	**The number of ** **involved lymph**	**Mean vitamin D ** **level (ng/ml) ± SD**	**p-value**
**Premenopause**	0	22.03 ± 15.375	0.65
	1 -3	23.25 ± 14.14	
	4 - 9	19.32 ± 11.75	
	>9	17.69 ± 10.89	
**Postmenopause**	0	22.72 ± 14.692	0.05
	1 -3	21.67 ± 14.10	
	4 - 9	23.12 ± 14.85	
	> 9	15.52 ± 9.48	

**Table 4:** The relationship between vitamin D level and stage of the disease in patients before and after menopause

**Menopausal status**	**Stage of the ** **disease**	**Mean vitamin ** **D level (ng/ml) ** **± SD**	**p-value**
**Premenopause**	1	25.73 + 13.785	0.231
	2a	21.53 + 16.106	
	2b	23.54 + 13.930	
	3a	20.79 + 11.761	
	3b	14.41 + 9.536	
	3c	30.50 + 3.536	
	4	9.88 + 5.666	
**Postmenopause**	1	25.82 + 15.719	0.04
	2a	26.77 + 11.316	
	2b	26.54 + 18.396	
	3a	26.93 + 13.920	
	3b	18.39 + 10.428	
	3c	6.50 + 0.00	
	4	13.53 + 11.901	

Serum vitamin D levels were not significantly different between the two groups. Tumor characteristics and their relation to vitamin D levels were evaluated separately in two groups of patients (pre-and post-menopause).

A significant relationship was observed between low levels of vitamin D, stage of the disease and tumor size.

**Table 5:** The relationship between vitamin D level and stage of the disease (early and advanced) after taking account of other independent variables

	**Mean**	**95% CI**	**P-value**					
			**Model 1**	**Model 2**	**Model 3**	**Model 4**	**Model 5**	**Model 6**
**Early **	24.6	22.1 to 27.0	0.002	0.001	0.005	0.004	0.026	0.003
**Advanced**	17.9	14.9 to 21.2						
Model 1: Independent t-test								
Model 2: ANCOVA (Age, BMI)								
Model 3: ANCOVA (ER, PR, HER2)								
Model 4: ANCOVA (Age, BMI, ER, PR, HER2)								
Model 5: ANCOVA (Age, BMI, ER, PR, HER2, Menopausal status)								
Model 6: ANCOVA (Age, BMI, ER, PR, HER2, Menopausal status, number of LN )								

Metastasis was only found in postmenopausal patients, but in premenopausal patients there was no significant association. Hence, our results indicate a significant relationship between low vitamin D levels and advanced stages of breast cancer in menopausal patients. The study of Palmieri et al.^15^ also showed that vitamin D levels in metastatic and advanced stages of breast cancer were significantly lower than early stages of the disease. However, based on study of Asvadi et al.^16^ only P53 gene alteration was associated with low vitamin D levels among the prognostic features of breast tumors. Tumor size, tumor grade, lymphnode involvement, disease stage and hormone receptor status were not associated with vitamin D levels. Larsson et al.^17^ reported a relationship between low levels of vitamin D and negative hormone receptors (ER- and PR-), but no relationship was reported between low levels of vitamin D and other features of tumor. The study of Hatse et al.^18^ also showed lower levels of vitamin D in patients with breast cancer compared to healthy controls.

The survey also revealed that only tumor size among the prognostic features of tumor had a significant correlation with lower levels of vitamin D and other tumor characteristics including tumor grade, receptor status and disease stage were not significantly associated with vitamin D level. A three-year follow-up study showed that higher levels of vitamin D had a significant relationship with improved overall survival, especially in breast cancer.

In a study in South Korea, a significant association was found between low levels of vitamin D and poor outcome in breast cancer and triple negative tumors.^19^ The study of Peppon et al.^20^ has shown a relationship between low serum levels of vitamin D and increased risk of estrogen- receptor negative (ER-) breast cancer. By contrast, the study conducted by Imtiaz et al.^21^ showed no relationship between serum vitamin D levels and tumor prognostic features. The fundamental importance of vitamin D is shown in regulating cell behaviors such as cell growth in in vitro studies of breast cancer cells as well as in vivo animal experiments^6^^,^^26^ as the ability of 1, 25 –hydroxy vitamin D in inhibiting proliferation and promoting differentiation and apoptosis.^27^^-^^30^

In addition, a descriptive study indicated lower levels of 1, 25 (OH) vitamin D in women with a primary diagnosis of breast cancer compared to healthy controls.^31^ The study also revealed a decreased serum vitamin D levels in patients with metastatic cancer of bone in comparison with early stages of the disease.^32^ It is not clear exactly why 25-hydroxy vitamin D levels in patients with advanced breast cancer are lower than those of early stages.

It is also not clearly identified in the research that low levels of vitamin D are the causes of the advanced stages of cancer or direct results of advanced disease such as reduced food intake due to cancer or impaired vitamin D synthesis in the skin. 24 hydroxylase, an enzyme that inactivate 1, 25 hydroxy vitamin D and plays a role in homeostasis of its serum level, appears to be oncogene.^33^ It has been shown that the incidence of 24- hydroxylase in primary tumors of breast is higher than normal breast tissue, and the level of 1, 24 and 25- hydroxyvitamin D is significantly higher in malignant breast tumors; but it does not cause anti-proliferative response in breast cancer cells. In vitro studies have shown that the effect of this enzyme can be reversible by antisense inhibition. Therefore, theoretically, there can be a potential mechanism for resistance to the effects of vitamin D on breast tumors (by dysregulation of 24-hydroxylase activity). However, it is not clear that how this mechanism is different in early and advanced stages of cancer.^34^ It is possible that higher dysregulation of vitamin D metabolism at the advanced stages of breast cancer leads to some paracrine effects of tumor; or tumors with high levels of 24-hydroxylase may be at higher risk of more advanced stages. On the other hand, microarray analysis revealed the existence of several key genes which are up- and down- regulated by vitamin D therapy. Cyclin-dependent kinase inhibitor P21 is one of the key genes that is upregulated due to treatment with vitamin D and has an important role in controlling the cell cycle.^35^ Thus; it appears that changes in serum levels of vitamin D could potentially result in significant effects on gene transcription and thus, on cell phenotype.

## CONCLUSION

 According to our results, it seems that lower levels of vitamin D are associated with worse clinicopathologic features. Thus, treatment of vitamin D deficiency in post-menopausal patients may be of great consequence. We suggest further studies on genetic regulation of vitamin D metabolism in breast cancer patients.
